# Personalized Prescription of Chemotherapy Based on Assessment of mRNA Expression of *BRCA1, RRM1, ERCC1, TOP1, TOP2α, TUBβ3*, *TYMS*, and *GSTP1* Genes in Tumors Compared to Standard Chemotherapy in the Treatment of Non-Small-Cell Lung Cancer

**DOI:** 10.3390/jpm12101647

**Published:** 2022-10-04

**Authors:** Matvey M. Tsyganov, Evgeny O. Rodionov, Marina K. Ibragimova, Sergey V. Miller, Olga V. Cheremisina, Irina G. Frolova, Sergey A. Tuzikov, Nikolai V. Litviakov

**Affiliations:** 1 Department of Experimental Oncology, Cancer Research Institute, Tomsk National Research Medical Center, Russian Academy of Sciences, 5, Kooperativny Street, 634050 Tomsk, Russia; 2 Department of Thoracic Oncology, Cancer Research Institute, Tomsk National Research Medical Center, Russian Academy of Sciences, 12, Savinykh Street, 634050 Tomsk, Russia; 3 Biological Institute, National Research Tomsk State University, Lenin Avenue, 36, 634050 Tomsk, Russia; 4 Department of Endoscopy, Cancer Research Institute, Tomsk National Research Medical Center, Russian Academy of Sciences, 12, Savinykh Street, 634050 Tomsk, Russia; 5 Imaging Department, Cancer Research Institute, Tomsk National Research Medical Center, Russian Academy of Sciences, 12, Savinykh Street, 634050 Tomsk, Russia

**Keywords:** non-small-cell lung cancer, adjuvant chemotherapy, gene expression, individualized chemotherapy, metastasis-free survival, overall survival

## Abstract

Objectives: A growing body of evidence suggests the important role of chemosensitive gene expression in the prognosis of patients with lung cancer. However, studies on combined gene expression assessments for personalized prescriptions of chemotherapy regimens in patients have not yet been conducted. The aim of this work was to conduct a prospective study on the appointment of personalized chemotherapy in patients with non-small-cell lung cancer. Materials and methods: The present study analyzed 85 patients with lung cancer (stage IIB-IIIB). Within this group, 48 patients received individualized chemotherapy, and 37 patients received classical chemotherapy. In the individualized chemotherapy group, the mRNA expression levels of *ERCC1, RRM1*, *TUBB3*, *TYMS*, *TOP1*, *TOP2α*, *BRCA1*, and *GSTP1* in lung tissues were measured by quantitative real-time PCR (qPCR), and an individual chemotherapy regimen was developed for each patient according to the results. Patients in the classical chemotherapy group received the vinorelbine/carboplatin regimen. Survival analyses were performed using the Kaplan–Meier method. Prognostic factors of metastasis-free survival (MFS) and overall survival (OS) of patients were identified via Cox’s proportional hazards regression model. Results: MFS and OS were significantly better in the personalized chemotherapy group compared to the classic chemotherapy group (MFS, 46.22 vs. 22.9 months, *p* = 0.05; OS, 58.6 vs. 26.9 months, *p* < 0.0001). Importantly, the best metastasis-free survival rates in the group with personalized ACT were achieved in patients treated with the paclitaxel/carboplatin regimen. Based on an assessment of chemosensitivity gene expression in the tumors, the classical chemotherapy strategy also increased the risk of death (HR = 14.82; 95% CI: 3.33–65.86; *p* < 0.000) but not metastasis (HR = 1.95; 95% CI: 0.96–3.98; *p* = 0.06) compared to the group of patients with chemotherapy. Conclusions: The use of combined *ERCC1*, *RRM1*, *TUBB3*, *TYMS*, *TOP1*, *TOP2α*, *BRCA1*, and *GSTP1* gene expression results for personalized chemotherapy can improve treatment efficacy and reduce unnecessary toxicity.

## 1. Introduction

Adjuvant chemotherapy (ACT) is now considered the standard of care for patients with resected stage IB non-small-cell lung cancer (NSCLC), as well as for patients with resected stages II and IIIA lung cancer, with a higher 5-year survival rate compared to surgical treatment alone [1,2]. It was shown that cisplatin-based adjuvant chemotherapy improves rates of metastasis-free and overall survival only in patients with stage IB NSCLC who have undergone complete resection [3,4]. Five-year overall survival for primary lung cancer is approximately 80% in patients with stage I disease but decreases to <45% in patients with advanced lung cancer of stage II or higher [5]. Treatment of stage III NSCLC is still a difficult and controversial task, mainly due to the different degrees of prevalence of the primary tumors that combine into stage III cancer, according to the TNM classification. Traditionally, locally advanced cancer is divided into stage IIIA with a 24% 5-year survival and stage IIIB with a 9% 5-year survival and poor prognosis [6]. Despite the continuous improvements in surgical methods, there remains no noticeable trend towards improvements in survival data. In the vast majority of patients with stage III cancer (35–50%), progression occurs due to the development of distant metastases [6].

Consequently, the main trend in modern oncology is understanding molecular biological changes in tumors and searching for the associations of such changes with the efficacy of treatment and prognosis of the disease. For example, differences in the expression of certain markers can explain why tumors comparable in size, prevalence, histological structure, etc., differ in their aggressive courses and disease outcomes. Many researchers are presently studying the possibility of assessing the sensitivity of a tumor to certain chemotherapy drugs [7].

The differential expression/co-expression of several genes such as excision repair cross-complementing 1 (*ERCC1*), ribonucleotide reductase subunit M1 (*RRM1*), thymidylate synthase (*TYMS*), class III β-tubulin (*TUBβ3*), DNA topoisomerase I and II alpha (*TOP1* and *TOP2α*), glutathione S-transferase Pi 1 (*GSTP1*), etc. in tumor tissues is closely related to chemoresistance and prognosis in cancer patients. For example, high expression levels of *ERCC1* and *BRCA1*, which are crucial for DNA repair, negatively affect the efficacy of platinum drugs and are thought to be a major predictor of tumor responses to platinum-based chemotherapy [8,9]. *GSTP1* is an enzyme that catalyzes the detoxification pathways of platinum drugs to protect cells, including tumor cells [10]. In addition, it was found that overexpression of *GSTP1* is associated with cisplatin-induced chemoresistance and cytotoxicity [11].

It was shown that the level of expression of *RRM1*, which is the main target of gemcitabine, is negatively correlated with the efficacy of gemcitabine [12]. *TUBβ3* is thought to be a marker of taxane resistance, and high levels of this gene expression are associated with a low response rate in patients treated with taxane-containing regimens [13,14]. The expression level of *TYMS*, which is a central enzyme in the folate metabolic pathway and a major target for cytotoxic antifolate chemotherapy drugs such as 5-fluorouracil, capecitabine, and gemcitabine, is negatively associated with the efficacy of antimetabolic agents [15]. *TOP1* and *TOP2α* are nuclear enzymes involved in changing the topology of DNA and are the main molecular targets for various cytotoxic drugs, particularly anthracyclines. It was shown that the level of *TOP2α* expression is positively correlated with the efficacy of these chemotherapy drugs [16]. Numerous TOP inhibitors, including etoposide, adriamycin, and camptothecin, are now widely used in clinical practice [17,18].

Despite the fact that increasingly more data indicate the important role of the presented genes in assessing chemosensitivity, studies on combined assessment of the expression of *ERCC1*, *RRM1*, *TUBβ3*, *TYMS*, *TOP1*, *TOP2α*, *BRCA1*, and *GSTP1* genes for personalized chemotherapy regimens in patients with lung cancer have not yet been conducted. Thus, the aim of this work was to conduct a prospective study on the prescription of personalized chemotherapy in patients with non-small-cell lung cancer.

## 2. Materials and Methods

### 2.1. The Study Group

The present study involved 85 patients with NSCLC stage IIB–IIIB cancer featuring central or peripheral localization and a morphologically verified diagnosis, who were treated at the clinic of the Research Institute of Oncology of the Tomsk National Research Medical Center in 2010–2018. The study was conducted in accordance with the 1964 Declaration of Helsinki (amended in 2013) [19] and with the permission of the local ethics committee of the institute (Protocol No. 1 of 15 January 2016); all patients signed their informed consent for this study. All patients received 2 courses of neoadjuvant chemotherapy (NAC) according to the scheme of vinorelbine 25 mg/m^2^ (days 1 and 8)/carboplatin AUC 6 (on day 2), with an interval of 3 weeks and subsequent assessment of the effects. The effectiveness of NAC was assessed using the RECIST 1.1 scale. After NAC, an operation was performed on the patients (pneumonectomy or lobectomy).

Further, to calculate the effectiveness of the personalized prescription of adjuvant chemotherapy, the patients were divided into two groups. The historical control group (*n* = 37) included patients who, after surgery, underwent 3 courses of adjuvant chemotherapy according to the standard scheme of vinorelbine 25 mg/m^2^ (days 1 and 8)/carboplatin AUC 6 (on day 2). The study group consisted of 48 patients. The appointment of the ACT regimen was personalized depending on the expression parameters of gene markers of chemosensitivity. After surgery, these patients underwent adjuvant chemotherapy with platinum doublets according to the following schemes: vinorelbine 25 mg/m^2^ (1st and 8th days)/carboplatin AUC 6 (on day 2); doxorubicin 50 mg/m^2^/carboplatin AUC 6 (on day 2); gemcitabine 1250 mg/m^2^ (days 1 and 8)/carboplatin AUC 6 (on day 2); paclitaxel 175 mg/m^2^/carboplatin AUC 6 (on day 2); irinotecan (60 mg/m^2^ (days 1 and 8)/carboplatin AUC 6 (on day 2); vinorelbine 25 mg/m^2^ (1st and 8th days)/cisplatin (75 mg/m^2^); gemcitabine 1250 mg/m^2^ (1st and 8th days)/cisplatin (75 mg/m^2^); paclitaxel 175 mg/m^2^/cisplatin (75 mg/m^2^). The interval between each chemotherapy course was 3 weeks. Chemotherapy was carried out under satisfactory general conditions and laboratory parameters of the patients, without deviations from the norm. After chemotherapy in the adjuvant mode, the frequency and nature of complications in the compared groups were assessed. There were no statistically significant differences found in the number of complications (*p* > 0.05) (Table 1).

Thus, the applied regimens (personalized chemotherapy) did not cause severe complications and were satisfactorily tolerated by the patients, allowing the patients to be fully treated.

The main clinical and pathological parameters of the patients included in the study and their comparisons are presented in Table 2.

Surgical material after chemotherapy (tumor tissue, unchanged lung tissue, ~30–60 mm^3^) was used as the test material. Two samples of tumor tissue were morphologically confirmed for each patient. The tissues were placed in an RNAlater (Sigma, St. Louis, MO, USA) incubator for 24 h at room temperature and stored at –80 °C until RNA extraction.

### 2.2. RNA Extraction

RNA was isolated from samples of normal and tumorous tissue using an RNeasy Plus mini Kit (Qiagen, Hilden, Germany #51304), according to the manufacturer’s instructions.

The RNA concentration and purity were assessed using a NanoDrop 2000 instrument (Thermo Fisher, Waltham, MA, USA). The concentration varied between 100 and 500 ng/µL and, A_260_/A_280_ and A_260_/A_230_; the ratios were 1.85–2.05 and 1.80–2.08, respectively. RNA integrity was assessed using a TapeStation instrument and R6K ScreenTape kit (Agilent Technologies, Santa Clara, CA, USA). The RIN values were 6.6–9.2. The RNA was reverse-transcribed into cDNA using a RevertAid™ kit (Thermo Fisher, Waltham, MA, USA) according to the manufacturer’s instructions. 

### 2.3. Expression Profiling of the Chemosensitivity Genes

Expression profiling of the *BRCA1*, *RRM1*, *ERCC1*, *TOP1*, *TOP2a*, *TUBβ3*, *TYMS*, and *GSTP1* genes was carried out via quantitative real-time PCR (qPCR) using custom fluorescent-labelled probes and a RotorGene-6000 instrument (Corbett Research, Mortlake, NSW, Australia). qRT-PCR was performed in triplicate for each sample in a volume of 15 μL containing 250 lM dNTPs (Sibenzyme, Novosibirsk, Russia), 300 nM forward and reverse primers, a 200 nM probe, 2.5 mM MgCl_2_, 19 SE buffer (67 mM Tris–HCl pH 8.8 at 25 °C, 16.6 mM (NH_4_)_2_SO_4_, 0.01% Tween-20), 2.5U Hot Start Taq polymerase (Sibenzyme, Novosibirsk, Russia), and 50 ng of a cDNA template. Samples were heated for 10 min at 95 °C, followed by 40 cycles of amplification for 10 s at 95 °C and 20 s at 60 °C. Primer and probe (FAM-BHQ1) sequences were designed using Vector NTI Advance 11.5, Oligo 7.5 and the NCBI Nucleotide Database (https://www.ncbi.nlm.nih.gov/gene/ accessed on 13 September 2022) (Table 3). The mean expression level of each target gene was calculated for tumor tissue normalized to *GAPDH* (glyceraldehydes-3-phosphatedehydrogenase) and ACTB (actin beta). The average Ct (cycle threshold) was estimated for both the gene of interest and *GAPDH* and *ACTB*. Relative expression was evaluated using the Pfaffl method [20] and measured in arbitrary units.

### 2.4. Selection and Implementation of Chemotherapy Schemes

**Figure 1:**
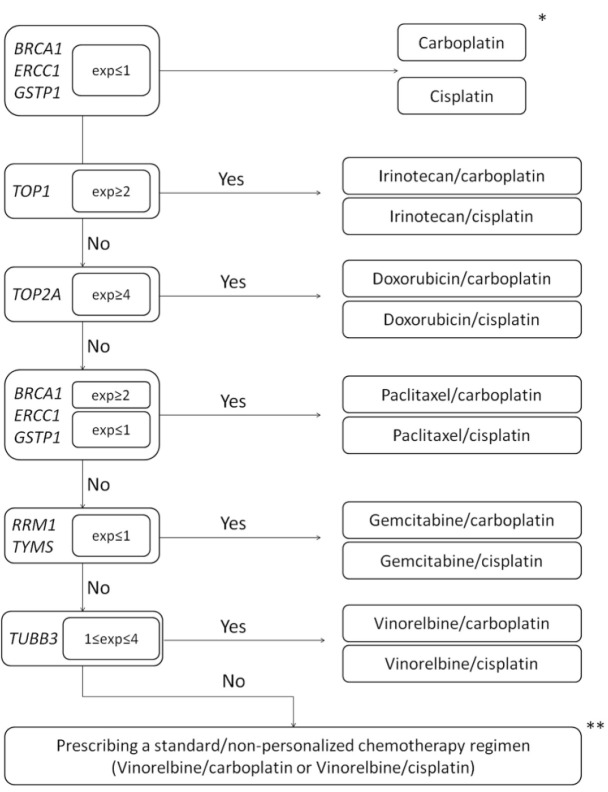
Algorithm for choosing a personalized regimen for adjuvant chemotherapy in patients with NSCLC, depending on the level of expression of chemosensitivity genes. Note: * According to international clinical guidelines, adjuvant chemotherapy is based on platinum-containing drugs, so the first drug in the regimen is cisplatin or carboplatin. ** If patients were unable to choose a personalized chemotherapy regimen, then such patients received a standard treatment regimen and were not included in the study.

The chemotherapy regimen for each patient in the personalized group was based on an assessment of the expression profiles of the genes for chemosensitivity. The principle for choosing chemotherapy drugs was as follows. Platinum drugs such as carboplatin and cisplatin were recommended for patients with low, absent, or moderate levels of *ERCC1*, *BRCA1,* and *GSTP1* gene expression [12]. It is important to note that, according to international clinical guidelines [21], adjuvant chemotherapy in patients with NSCLC is recommended at stages II and III and should be based on platinum-containing drugs. Therefore, cisplatin or carboplatin were prescribed individually in all cases (Figure 1).

Gemcitabine is recommended for patients with low *RRM1* and *TYMS* expression [12]. Anti-microtubule drugs, particularly paclitaxel, were prescribed when the level of *TUBβ3* expression was moderate and not prescribed when this gene was highly expressed [14]. Irinotecan and doxorubicin were prescribed depending on the expression of genes for topoisomerase I and II, respectively. Prescription of these drugs was recommended only with high expression of these genes (over 2 and 4, respectively) [22].

Notably, in our study, a choice in favor of carboplatin and/or cisplatin was made individually due to the pronounced nephro- and neurotoxicity and emetogenicity of cisplatin, as well as the better tolerability of carboplatin. Cisplatin has high renal toxicity, and when administered in high doses, it is extremely important to ensure increased urine output. To ensure sufficient urine output, before administration of the drug and within 24 h after injection, the patient was intravenously injected with a large amount of liquid. Considering these data, patients with chronic renal failure, those with heart failure, and those that underwent pulmonectomy were mainly prescribed carboplatin.

### 2.5. Statistical Analysis

Statistical analysis was performed using the Statistica 10.0 software (StatSoft Inc., Palo Alto, CA, USA). The Shapiro–Wilk criterion was used to assess the normality of the sample, and the arithmetic mean value and standard error were calculated for each sample group. The expression level of the studied genes was divided by quartiles using basic statistics. A Wilcoxon–Mann–Whitney test was used to assess the differences between studied groups. The comparison of the frequencies in qualitative data was analyzed using a two-tailed Fisher’s exact test. The 95% confidence intervals (95% CIs) were calculated using the exact method. All *p* values were two-tailed; a *p* value of 0.05 was considered significant. To analyze the overall (OS) and metastasis-free survival (MFS), survival curves were constructed using the Kaplan–Meier method. Comparison of the statistical significance of differences between groups was performed using a log-rank test. A chi-square test was used to assess differences in the frequencies of the study groups.

## 3. Results

None of the patients received targeted therapy or any additional treatment before or after surgery. There were no significant differences in baseline characteristics between the study groups of patients who received personalized and classical chemotherapy (Table 1). Statistically significant differences were found only in the nature of surgery. Among patients who received personalized treatment, lobectomy or bilobectomy prevailed in 79.2% (38/48 cases) (Fisher Exact Probability Test, *p* = 0.005). Whereas, in the control group, the surgery was more radical, and the frequencies of lobectomy and pneumonectomy were approximately equal (Table 2). Details of the baseline characteristics of the two groups of patients are presented in Table 2.

The median follow-up was 32.0 months (range 2–88 months) among patients included in the study. In the control group and main group, this figure was 27 months (2–55 months) and 48.0 months (2–88 months), respectively. An adjuvant chemotherapy regimen of vinorelbine/carboplatin was used for the control group. In total, 37 patients in the group received a standard chemotherapy regimen. Distant metastases developed in 17 (45.9%) patients within 2–32 months from the moment of diagnosis. One-year metastasis-free survival was 61.8%, 2-year survival was 58.8%, 3-year survival was 37.0%, and 4-year survival was 14.3%. In the group with a personalized approach for prescribing adjuvant chemotherapy, metastatic disease developed in 15 patients (31.3%) over a period of 2–73 months, and rates of 1-year, 2-year, and 5-year metastasis-free survival were 85.1%, 71.7%, and 61.6%, respectively.

Details of the chemotherapy regimens in the group with personalized ACT are presented in Table 4.

**Figure 2:**
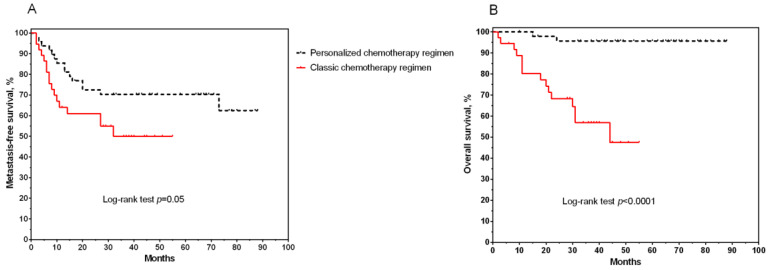
Curves of metastasis-free (**A**) and overall (**B**) survival of patients with non-small-cell lung cancer, depending on the choice of adjuvant chemotherapy regimen.

Further, metastasis-free and overall survival rates were assessed in patients of the study groups using the Kaplan–Meier method (Figure 2).

The median metastasis-free survival was 27 months (range 2–55) in the control group of patients and 48 months (range 2–88 months) in the group with personalized ACT. The average value of the rates was 46.22 ± 3.98 months in the group with personalized chemotherapy compared to 22.9 ± 2.65 months in the classical group (Figure 2A). Differences were statistically significant (log-rank test *p* = 0.05). Very good results were found for overall survival (Figure 2B). Patients with a personalized chemotherapy regimen had a 96% survival rate compared to the control group, where the lower limit was 48% (log-rank test *p* < 0.0001). At the same time, the average OS for the control group was 26.9 ± 2.39 months versus 58.6 ± 2.9 months in the second group of studied patients, and the median values were 29 months (2–55 months) and 65 months (10–88 months), respectively.

**Figure 3:**
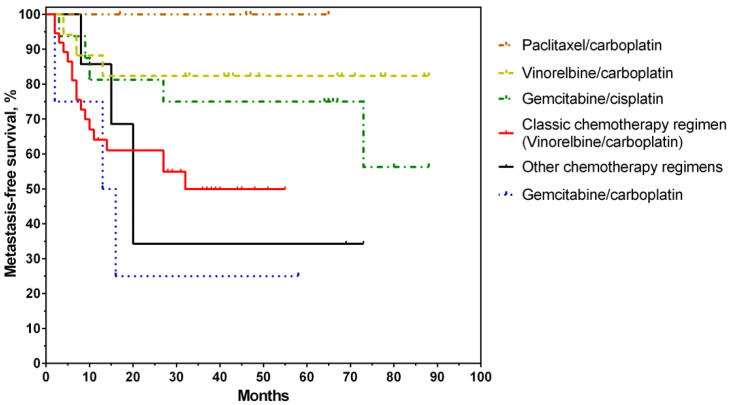
Metastasis-free survival curves of the study patients with non-small-cell lung cancer depending on the adjuvant chemotherapy regimen.

Notably, the best rates of metastasis-free survival in the group that received personalized ACT were achieved among patients treated with the paclitaxel/carboplatin regimen (Figure 3). These patients (8.3%, 4/48 cases) had a 100% survival rate.

Slightly lower rates of 5-year MFS (83%) were observed in the group of patients treated with the vinorelbine/carboplatin regimen (35.4%, 17/48 cases). These rates were about the same in the control group (50%) and the group with the personalized gemcitabine/cisplatin regimen (33.3%, 16/48 cases; MFS rate: 57%). The worst metastasis-free survival rates were achieved in the gemcitabine/carboplatin regimen (25%) (Figure 3). Metastatic disease developed in 3 of 4 patients within 2–16 months. Thus, the most promising regimens to be prescribed in personalized chemotherapy are paclitaxel/carboplatin, vinorelbine/carboplatin, and gemcitabine/carboplatin, subject to their personalized assignment.

In addition, a multivariate regression analysis was performed to identify prognostic factors for metastasis-free and overall survival (Table 5).

It was found that lymph node metastases (N_2_ patients) increased the risk of tumor metastasis (HR = 2.55; 95% CI: 1.23–5.28; *p* = 0.01). Stage IIIB cancer was also identified as an independent risk factor affecting both metastasis-free (HR = 3.32; 95% CI: 1.33–8.30; *p* = 0.01) and overall survival (HR = 6.84; 95% CI: 2.26–20.73; *p* = 0.001). Equally, performing an operation in the pneumonectomy mode increased the risk of death (HR = 7.43; 95% CI: 2.41–22.85; *p* < 0.000). The classical chemotherapy strategy is also a factor found to increase the risk of death (HR = 14.82; 95% CI: 3.33–65.86; *p* < 0.000) but not metastasis (HR = 1.95; 95% CI: 0.96–3.98; *p* = 0.06), compared to the group of patients who received chemotherapy and based on assessments of the chemosensitivity gene expression in the tumors (Table 5).

An age of patients less than 50 years is a favorable factor for overall survival rates (HR = 0.95; 95% CI: 0.45–2.00; *p* = 0.05).

## 4. Discussion

A personalized approach to prescribing chemotherapy is now actively used in modern oncology. At the same time, assessing the expression of chemosensitivity genes that determine the sensitivity of tumor cells to certain chemotherapy drugs and the appointment of a chemotherapy regimen depending on the level of expression represents a promising direction [23]. Our study showed that, in general, the use of a personalized approach in prescribing postoperative chemotherapy improved metastatic and overall survival compared to the historical control group. However, at the same time, it is important to note that some (personalized) chemotherapy regimens had low survival rates, possibly due to the small sample of patients treated with such regimens (for example, Vinorelbine/cisplatin or Paclitaxel/carboplatin). This result may be due to the fact that, for example, the *TOP1* gene is rarely overexpressed, meaning that there were very few patients on the Irinotecan/carboplatin chemotherapy regimen. The study is currently ongoing, and the number of patients with rare regimens will increase over time.

It was found that high expression of *ERCC1* (excision repair gene product) is associated with resistance to platinum-based chemotherapy [24]. *BRCA1* gene overexpression is also associated with a low efficacy of cisplatin chemotherapy, as well as low rates of metastasis-free and overall survival [25]. Low *RRM1* expression is a predictor of high survival with gemicitabine-based chemotherapy [25]. High expression of the thymidylate synthase enzyme was found to be statistically significantly correlated with sensitivity to gemcitabine [26] because thymidylate synthase participates in the de novo formation of thymidylate, a precursor of thymidine triphosphate that serves as a nucleotide necessary for DNA synthesis. In addition, *TYMS* is a major target for antifolate cytotoxic drugs such as 5-fluorouracil and capecitabine. This enzyme exerts antitumor effects by inhibiting deoxythymidylate synthesis and additionally influencing the synthesis and repair of DNA [27]. High expression of β3-tubulin (*TUBβ3*) is associated with resistance to docetaxel and paclitaxel [28]. The expression of topoisomerase group genes (topoisomerase I (*TOP1*) and II alpha (*TOP2α*)) is important for doxorubicin [29]. These enzymes change the topology of DNA and catalyze the unwinding of DNA superspirals, as well as the breaking and cross-linking of nucleic acid molecules.

To date, there are few studies on the use of a comprehensive approach for assessing chemosensitivity gene expression as a predictive or prognostic marker. Recently, the expression of genes such as *TYMS*, *RRM1*, *TUBβ3*, and *EGFR* was found to be associated with tumor histological type (at *p* < 0.05) and progression-free survival rates. It was found that patients are treated with platinum-based drugs with a low expression of *RRM1*, when combining low expression of *RRM1* and *ERCC1*, patients present higher survival rates (*p* < 0.05) [30]. Interesting data were previously obtained for the presented genes in breast cancer. In a 2020 study, the authors showed that the use of personalized chemotherapy, based on the assessment of gene expression, is an independent factor for increasing recurrence-free survival (HR = 0.389, 95% CI: 0.153–0.989, *p* = 0.047) but not overall survival (HR = 0.340, 95% CI: 0.107–1.078, *p* = 0.067) [31]. It was found that the combined evaluation of *ERCC1*, *RRM1*, *TUBβ3*, *TYMS*, and *TOP2α* gene expression can increase the effectiveness of treatment and reduce toxicity from chemotherapy. A recent study showed that *TOP2a* can serve as a good prognostic factor in the treatment of patients with NSCLC [16]. Patients with higher levels of expression had lower recurrence-free survival compared to *TOP2α*-negative patients.

In a previous meta-analysis, it was found that sensitivity to chemotherapy with platinum drugs among patients without *ERCC1* expression in the middle and late stages of NSCLC was better than in patients with positive expression (*p* < 0.01) [32]. In addition, resistance to platinum doublets of chemotherapy was found to be greatly increased in patients with a KRAS mutation and low expression of the *BRCA1* and *TYMS* genes in tumor tissue [33]. In a study evaluating *EGFR* and *KRAS* gene mutations along with the mRNA expression levels of *ERCC1*, *TUBβ3*, *TYMS*, *RRM1*, and *EGFR*, it was shown that when using a personalized approach for prescribing neoadjuvant chemotherapy according to the docetaxel/platinum regimen, the response rate was 13.3% (4/30 cases) for complete regression, 63.3% (19/30 cases) for stabilization, and 23.4% (7/30 cases) for tumor progression. In the group that received a chemotherapy regimen of gemcitabine/platinum, the response rate was one patient (12.5%) with complete tumor regression, five patients with stabilization (62.5%), and two patients with tumor progression (25%) [34]. Notably, the personalized prescription of a conventional chemotherapy regimen for patients with NSCLC has made it possible to achieve much greater efficiency in terms of increasing patient survival compared to the use of many new targeted drugs.

## 5. Conclusions

Ultimately, a personalized approach to prescribing an adjuvant chemotherapy regimen significantly increased the survival rates of patients with NSCLC. This result demonstrates that the reserves of conventional chemotherapy, in terms of increasing the effectiveness of treatment among patients with NSCLC, are far from being exhausted and that a personalized approach will significantly optimize treatment and increase survival. Nevertheless, future studies are needed on the associations between chemosensitivity gene expression and the search for new predictive markers of the sensitivity and resistance of lung tumors to conventional chemotherapy.

## Figures and Tables

**Table 1:** The frequency and nature of adverse events during chemotherapy in patients with non-small-cell lung cancer.

Complication		Number of Patients		*p*-Value
		Control Group (*n* = 37)	Main Group (*n* = 48)	
Anemia	1–2 degrees	8 (21.6)	8 (16.7)	1.00
	3–4 degrees	2 (5.4)	2 (4.2)	
Leukopenia	1–2 degrees	8 (21.6)	8 (16.7)	1.00
	3–4 degrees	2 (5.4)	2 (4.2)	
Thrombocytopenia	1–2 degrees	4 (10.8)	7 (14.6)	1.00
	3–4 degrees	1 (2.7)	1 (2.1)	
Hepatotoxicity		7 (18.9)	11 (22.9)	1.00
Nephrotoxicity		1 (2.7)	2 (4.2)	1.00
Nausea, vomiting		6 (16.2)	6 (12.5)	1.00
Arthralgia/myalgia		5 (13.5)	12 (25.0)	1.00

**Table 2:** Clinical and pathological characteristics of non-small-cell lung cancer patients.

Clinical and Pathological Parameter		Number of Patients		*p*-Value
		Control Group (*n* = 37)	Main Group (*n* = 48)	
Gender	Male	6 (16.2)	6 (12.5)	0.75
	Female	31 (83.8)	42 (87.5)	
Age	Average	57.2 ± 0.99	59.0 ± 1.02	0.12
	≤50 years	7 (18.9)	7 (14.6)	0.76
	>50 years	30 (81.1)	41 (85.4)	
Tumor size	T_1_	0 (0.0)	4 (8.3)	0.18
	T_2_	6 (16.2)	11 (23.0)	
	T_3_	24 (64.9)	28 (58.3)	
	T_4_	7 (18.9)	5 (10.4)	
Lymphogenous metastasis	N_0_	12 (32.4)	13 (27.1)	0.77
	N_1_	13 (35.1)	19 (39.6)	
	N_2_	10 (27.0)	15 (31.3)	
	N_3_	2 (5.5)	1 (2.1)	
TNM stage	IIB	12 (32.4)	13 (27.1)	0.75
	IIIA	21 (56.8)	31 (64.6)	
	IIIB	4 (10.8)	4 (8.3)	
Clinical and anatomical form	Peripheral	18 (48.6)	21 (43.8)	0.66
	Central	19 (51.4)	27 (56.3)	
Histological type of the tumor	Squamous cell carcinoma	23 (62.2)	35 (72.9)	0.35
	Adenocarcinoma	14 (37.8)	13 (27.1)	
Effect of NAC	Full regression	1 (2.7)	1 (2.1)	0.10
	Partial regression	7 (18.9)	20 (41.7)	
	Stabilization	29 (78.4)	26 (54.1)	
	Progression	0 (0.0)	1 (2.1)	
Nature of surgery	Pneumonectomy	19 (51.4)	10 (20.8)	**0.005**
	Lobectomy	18 (48.6)	38 (79.2)	
ACT scheme	Vinorelbine/carboplatin	37 (100.0)	17 (35.4)	-
	Vinorelbine/cisplatin	-	2 (4.2)	
	Gemcitabine/carboplatin	-	4 (8.3)	
	Gemcitabine/cisplatin	-	16 (33.3)	
	Paclitaxel/carboplatin	-	4 (8.3)	
	Paclitaxel/cisplatin	-	1 (2.1)	
	Doxorubicin/carboplatin	-	3 (6.3)	
	Irinotecan/carboplatin	-	1 (2.1)	
Hematogenous metastasis	Yes	17 (45.9)	15 (31.3)	0.18
	No	20 (54.1)	33 (68.8)	

Note: Statistically significant differences are in bold.

**Table 3:** The sequences of primers and probes of genes.

Gene	Amplicon (bp)	Sequence
*GAPDH*	124 bp	F 5′-gccagccgagccacatc-3′
		R 5′-ggcaacaatatccactttaccaga-3′
		Probe 5′-cgcccaatacgaccaaatccg-3′
*ACTB*	73 bp	F 5′-gagaagatgacccagatcatgtt-3′
		R 5′-atagcacagcctggatagcaa-3′
		Probe 5′-agaccttcaacaccccagccat-3′
*RRM1*	94 bp	F 5′-actaagcaccctgactatgctatcc-3′
		R 5′-cttccatcacatcactgaacacttt-3′
		Probe 5′-cagccaggatcgctgtctctaacttgca-3′
*ERCC1*	121 bp	F 5′-ggcgacgtaattcccgacta-3′
		R 5′-agttcttccccaggctctgc-3′
		Probe 5′-accacaacctgcacccagactacatcca-3′
*BRCA1*	107 bp	F 5′-acagctgtgtggtgcttctgtg-3′
		R 5′-cattgtcctctgtccaggcatc-3′
		Probe 5′-catcattcacccttggcacaggtgt-3′
*TOP1*	97 bp	F 5′-ggcgagtgaatctaaggataatgaa-3′
		R 5′-tggatatcttaaagggtacagcgaa-3′
		Probe 5′-accattttcccatcatcctttgttctgagc-3′
*TOP2α*	75 bp	F 5′-agtcgctttcagggttcttgag-3′
		R 5′-tttcatttacaggctgcaatgg-3′
		Probe 5′-cccttcacgaccgtcaccatgga-3′
*TUBΒ3*	71 bp	F 5′-gggccaagttctgggaagtc-3′
		R 5′-cgagtcgcccacgtagttg-3′
		Probe 5′-atgagcatggcatcgaccccagc-3′
*TYMS*	91 bp	F 5′-tctggaagggtgttttgga-3′
		R 5′-tcccagattttcactccctt-3′
		Probe 5′-tctttagcatttgtggatcccttga-3′
*GSTP1*	84 bp	F 5′-ctggtggacatggtgaatgac-3′
		R 5′-cttgcccgcctcatagttg-3′
		Probe 5′-aggacctccgctgcaaatacatctc-3′

Note: All Probes: FAM→BHQ1; bp, base pair; F, forward primer; R, reverse primer.

**Table 4:** Chemotherapy regimens for the studied groups of patients with non-small-cell lung cancer.

	No. of Cycles				
Chemotherapy Regimens	1	2	3	4	n
Personalized chemotherapy					
Vinorelbine (25 mg/m^2^)/carboplatin (AUC 6)	1	2	10	4	17
Vinorelbine (25 mg/m^2^)/cisplatin (75 mg/m^2^)				2	2
Gemcitabine (1250 mg/m^2^)/carboplatin (AUC 6)		3	1		4
Gemcitabine (1250 mg/m^2^)/cisplatin (AUC 6)	3	2	10	1	16
Paclitaxel (175 mg/m^2^)/carboplatin (AUC 6)	2	1		1	4
Paclitaxel (175 mg/m^2^)/cisplatin (75 mg/m^2^)			1		1
Doxorubicin (50 mg/m^2^)/carboplatin (AUC 6)			3		3
Irinotecan (75 mg/m^2^)/carboplatin (AUC 6)			1		1
Classic chemotherapy					
Vinorelbine (25 mg/m^2^)/carboplatin (AUC 6)	3	17	15	2	37

**Table 5:** Multivariate Cox regression analysis for metastasis-free and overall survival of patients in the study groups.

Factor	MFS		OS	
	HR (95% CI)	*p*-Value	HR (95% CI)	*p*-Value
Gender				
Male	1.00		1.00	
Female	0.78 (0.43–1.42)	0.43	0.95 (0.45–2.00)	0.90
Age				
>50	1.00		1.00	
≤50	1.13 (0.66–1.90)	0.64	0.59 (0.35–1.00)	**0.05**
Tumor size				
T_1–2_	1.00		1.00	
T_3–4_	1.07 (0.48–2.38)	0.86	5.95 (0.78–44.93)	0.08
Lymphogenous metastasis				
N_0_	1.00		1.00	
N_1_	0.60 (0.22–1.62)	0.32	0.92 (0.24–3.44)	0.90
N_2_	2.55 (1.23–5.28)	**0.01**	2.00 (0.74–5.41)	0.16
N_3_	2.69 (0.63–11.39)	0.17	2.53 (0.33–19.29)	0.37
TNM stage				
IIB	1.00		1.00	
IIIA	1.50 (0.62–3.60)	0.35	1.40 (0.37–5.17)	0.61
IIIB	3.32 (1.33–8.30)	**0.01**	6.84 (2.26–20.73)	**0.001**
Clinical and anatomical form				
Central	1.00		1.00	
Peripheral	0.99 (0.49–1.98)	0.97	0.62 (0.23–1.69)	0.35
Histological type of the tumor				
Squamous cell carcinoma	1.00		1.00	
Adenocarcinoma	0.65 (0.29–1.46)	0.30	0.66 (0.21–2.05)	0.48
Effect of NAC				
Full/partial regression	1.00		1.00	
Stabilization/progression	1.87 (0.83–4.19)	0.12	47.25 (0.84–2630.40)	0.06
Nature of surgery				
Lobectomy	1.00		1.00	
Pneumonectomy	1.45 (0.71–2.94)	0.30	7.43 (2.41–22.85)	**<0.000**
Chemotherapy strategy				
Personalized chemotherapy regimen	1.00		1.00	
Classic chemotherapy regimen	1.95 (0.96–3.98)	0.06	14.82 (3.33–65.86)	**<0.000**

Note: All Probes: FAM→BHQ1; bp, base pair; F, forward primer; R, reverse primer.

## Data Availability

Database No. 2018620216 dated 6 February 2018 “Database of the outcome of patients diagnosed with non-small cell lung cancer, taking into account standard and/or personalized adjuvant chemotherapy” Tsyganov M.M., Rodionov E.O., Deryusheva I.V., Ibragimova M.K., Efteev L.A., Miller S.V., Tuzikov S.A., Litvyakov N.V. Database No. 2018621741 dated 7 November 2018 “Database of the outcome of patients diagnosed with non-small cell lung cancer with a personalized approach to adjuvant chemotherapy after the radical surgical stage of treatment”, Deryusheva I.V., Efteev L.A., Rodionov E.O., Tsyganov M.M., Ibragimova M.K., Pevzner A.M., Miller S.V., Tuzikov S.A., Litvyakov N.V.
